# Crystal structure of 5-bromo-2,4,6-trimethyl-3-[(2-methyl­phen­yl)sulfin­yl]-1-benzo­furan

**DOI:** 10.1107/S2056989015013687

**Published:** 2015-07-25

**Authors:** Hong Dae Choi, Uk Lee

**Affiliations:** a Department of Chemistry, Dongeui University, San 24 Kaya-dong, Busanjin-gu, Busan 614-714, Republic of Korea; b Department of Chemistry, Pukyong National University, 599-1 Daeyeon 3-dong, Nam-gu, Busan 608-737, Republic of Korea

**Keywords:** crystal structure, benzo­furan, π–π inter­actions, C—H⋯π inter­actions, C—H⋯O hydrogen bonds, Br⋯Br contacts

## Abstract

In the title compound, C_18_H_17_BrO_2_S, the dihedral angle between the mean planes of the benzo­furan [r.m.s. deviation = 0.025 (2) Å] and the 2-methyl­benzene rings is 87.87 (5)°. In the crystal, mol­ecules are linked into supra­molecular layers parallel to (0-11) by C—H⋯O hydrogen bonds and Br⋯Br [3.4521 (5) Å] contacts. These are connected into a three-dimensional architecture *via* C—H⋯π inter­actions, which link inversion-related mol­ecules into dimers, and π–π inter­actions between the benzene and furan rings [centroid–centroid distance = 3.573 (2) Å].

## Related literature   

For the pharmacological properties of benzo­furan compounds, see: Aslam *et al.* (2009 ▸); Galal *et al.* (2009 ▸); Howlett *et al.* (1999 ▸); Wahab Khan *et al.* (2005 ▸); Ono *et al.* (2002 ▸). For a related structure, see: Choi *et al.* (2014 ▸). For synthetic details, see: Choi *et al.* (1999 ▸).
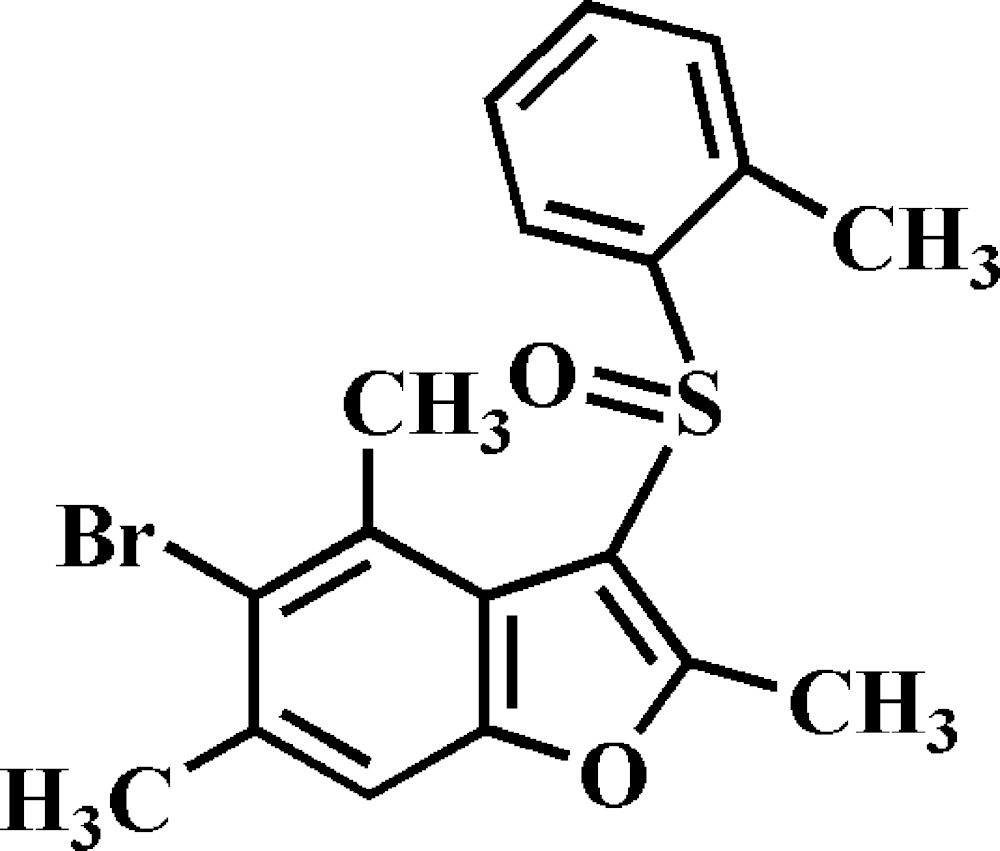


## Experimental   

### Crystal data  

C_18_H_17_BrO_2_S
*M* *_r_* = 377.29
Triclinic, 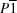

*a* = 7.4011 (2) Å
*b* = 10.6609 (2) Å
*c* = 11.1857 (2) Å
α = 67.265 (1)°
β = 86.593 (1)°
γ = 79.384 (1)°
*V* = 800.00 (3) Å^3^
*Z* = 2
Mo *K*α radiation
μ = 2.70 mm^−1^
*T* = 173 K
0.60 × 0.54 × 0.48 mm

### Data collection  

Bruker SMART APEXII CCD diffractometer
Absorption correction: multi-scan (*SADABS*; Bruker, 2009 ▸) *T*_min_ = 0.269, *T*_max_ = 0.746
14745 measured reflections
3982 independent reflections
3297 reflections with *I* > 2σ(*I*)
*R*_int_ = 0.046

### Refinement  

*R*[*F* ^2^ > 2σ(*F* ^2^)] = 0.035
*wR*(*F* ^2^) = 0.087
*S* = 1.05
3982 reflections
204 parameters
H-atom parameters constrained
Δρ_max_ = 0.48 e Å^−3^
Δρ_min_ = −0.79 e Å^−3^

### 

Data collection: *APEX2* (Bruker, 2009 ▸); cell refinement: *SAINT* (Bruker, 2009 ▸); data reduction: *SAINT*; program(s) used to solve structure: *SHELXS97* (Sheldrick, 2008 ▸); program(s) used to refine structure: *SHELXL2014* (Sheldrick, 2015 ▸); molecular graphics: *ORTEP-3 for Windows* (Farrugia, 2012 ▸) and *DIAMOND* (Brandenburg,1998 ▸); software used to prepare material for publication: *SHELXL2014*.

## Supplementary Material

Crystal structure: contains datablock(s) I. DOI: 10.1107/S2056989015013687/tk5377sup1.cif

Structure factors: contains datablock(s) I. DOI: 10.1107/S2056989015013687/tk5377Isup3.hkl

Supporting information file. DOI: 10.1107/S2056989015013687/tk5377Isup3.cml

**Fig. 1.**
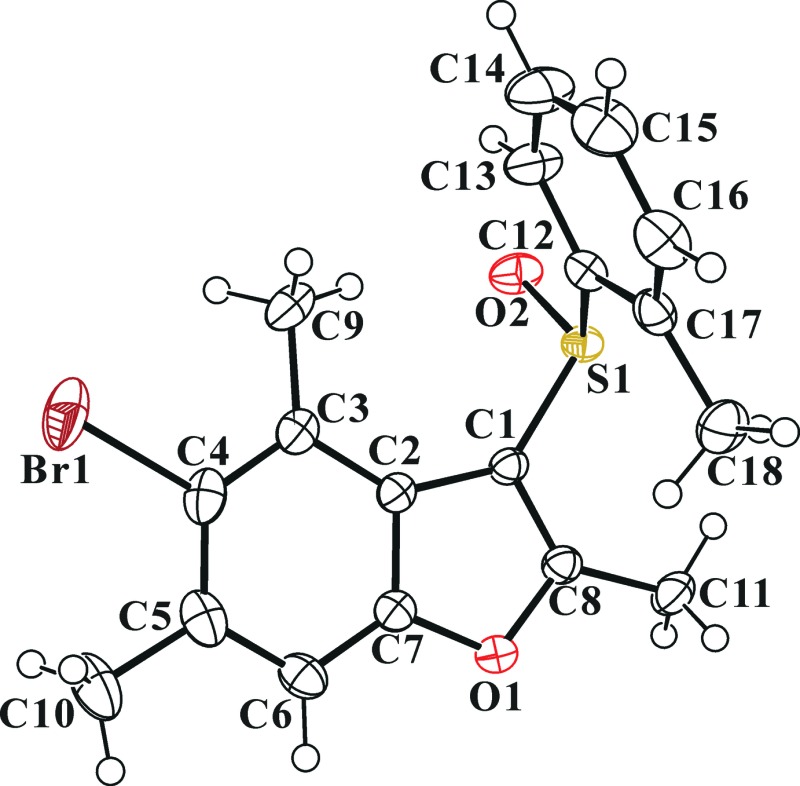
The molecular structure of the title compound with the atom numbering scheme. Displacement ellipsoids are drawn at the 50% probability level. H atoms are presented as small spheres of arbitrary radius.

**Fig. 2.**
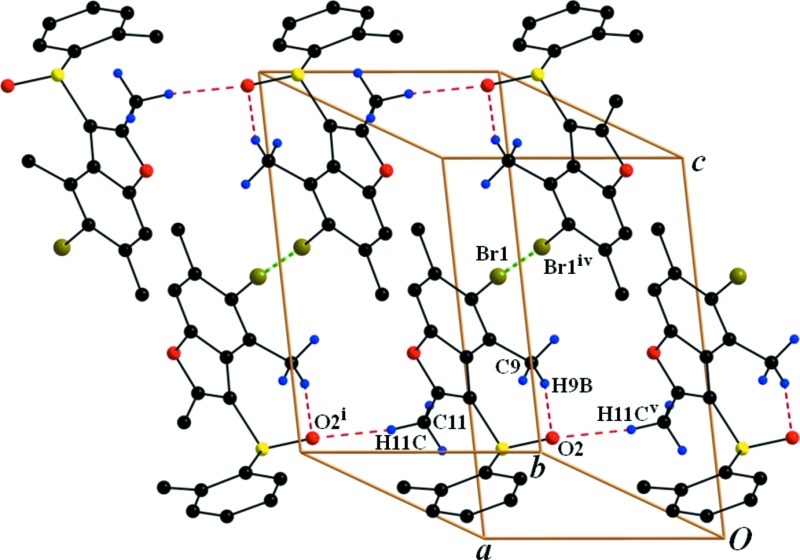
A view of the C—H···O and Br···Br interactions (dotted lines) in the crystal structure of the title compound. H atoms non-participating in hydrogen-bonding were omitted for clarity. [Symmetry codes: (i) x + 1, y, z; (iv) - x, - y + 2, - z + 1; (v) x - 1, y, z.]

**Fig. 3.**
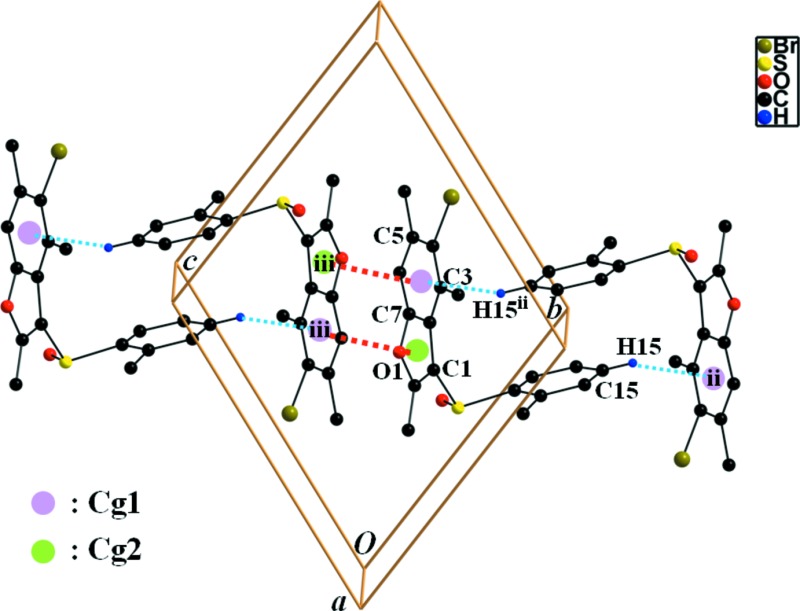
A view of the C—H···π and π–π interactions (dotted lines) in the crystal structure of the title compound. H atoms non-participating in hydrogen-bonding were omitted for clarity. [Symmetry codes: (ii) - x + 1, - y + 2, - z; (iii) - x + 1, - y + 1, - z + 1.]

. DOI: 10.1107/S2056989015013687/tk5377fig1.tif
The mol­ecular structure of the title compound with the atom numbering scheme. Displacement ellipsoids are drawn at the 50% probability level. H atoms are presented as small spheres of arbitrary radius.

x y z x y z x y z . DOI: 10.1107/S2056989015013687/tk5377fig2.tif
A view of the C—H⋯O and Br⋯Br inter­actions (dotted lines) in the crystal structure of the title compound. H atoms non-participating in hydrogen-bonding were omitted for clarity. [Symmetry codes: (i) *x* + 1, *y*, *z*; (iv) − *x*, − *y* + 2, − *z* + 1; (v) *x* − 1, *y*, *z*.]

x y z x y z . DOI: 10.1107/S2056989015013687/tk5377fig3.tif
A view of the C—H⋯π and π–π inter­actions (dotted lines) in the crystal structure of the title compound. H atoms non-participating in hydrogen-bonding were omitted for clarity. [Symmetry codes: (ii) − *x* + 1, − *y* + 2, − *z*; (iii) − *x* + 1, − *y* + 1, − *z* + 1.]

CCDC reference: 1413623

Additional supporting information:  crystallographic information; 3D view; checkCIF report

## Figures and Tables

**Table 1:** Hydrogen-bond geometry (, ) *Cg*1 is the centroid of the C2C7 benzene ring.

*D*H*A*	*D*H	H*A*	*D* *A*	*D*H*A*
C9H9*B*O2	0.98	2.33	3.274(3)	162
C11H11*C*O2^i^	0.98	2.32	3.276(2)	166
C15H15*Cg*1^ii^	0.95	2.88	3.659(3)	140

Symmetry codes: (i) 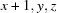; (ii) 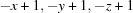.

## supplementary crystallographic information

### S1. Comment

Many compounds involving a benzofuran skeleton show interesting pharmacological properties such as anti-bacterial, anti-fungal, anti-tumour and anti-viral activities (Aslam *et al.*, 2009; Galal *et al.*, 2009; Wahab Khan *et al.*, 2005), and potential inhibitor of *β*-amyloid aggregation (Howlett *et al.*, 1999; Ono *et al.*, 2002). As a part of our continuing project on benzofuran derivatives (Choi *et al.*, 2014), we report herein the crystal structure of the title compound. In the title molecule (Fig. 1), the benzofuran unit is essentially planar, with a mean deviation of 0.025 (2) Å from the least-squares plane defined by the nine constituent atoms. The dihedral angle formed by the benzofuran ring and the 2-methylbenzene ring is 87.87 (5) Å. In the crystal structure (Fig. 2), molecules are linked by C—H···O hydrogen bonds (Table 1) and Br1···Br1^iv^ [3.4521 (5) Å] contacts in the (0 -1 1) plane. Further, inversion-related molecules are paired into dimers *via* C—H···π interactions (Fig. 3, Table 1, Cg1 is the centroid of the C2–C7 benzene ring). These dimers are further linked by π–π interactions between the benzene and furan rings of neighbouring molecules, with a Cg1···Cg2^ii^ distance of 3.573 (1) Å and an interplanar distance of 3.488 (2) Å resulting in a slippage of 0.775 (2) Å (Cg2 is the centroid of the C1/C2/C7/O1/C8 furan ring).

### S2. Experimental

The starting material 5-bromo-2,4,6-trimethyl-3-(2-methylphenylsulfanyl)-1-benzofuran was prepared by the literature method (Choi *et al.*, 1999). 3-Chloroperoxybenzoic acid (77%, 224 mg, 1.0 mmol) was added in small portions to a stirred solution of 5-bromo-2,4,6-trimethyl-3-(2-methylphenylsulfanyl)-1-benzofuran (285 mg, 0.9 mmol) in dichloromethane (25 ml) at 273 K. After being stirred at room temperature for 8 h, the mixture was washed with a saturated sodium bicarbonate solution (2 x 10 ml). The organic layer was separated, dried over magnesium sulfate, filtered and concentrated at reduced pressure. The residue was purified by column chromatography (hexane–ethyl acetate, 1:1 *v*/*v*) to afford the title compound as a colourless solid [yield 68% (226 mg); m.p.: 466–467 K; *R*_f_ = 0.51 (hexane–ethyl acetate, 1:1 *v*/*v*)]. Single crystals suitable for X-ray diffraction were prepared by slow evaporation of a solution of the title compound (21 mg) in ethyl acetate (20 ml) at room temperature.

### S3. Refinement

All H atoms were positioned geometrically and refined using a riding model, with C—H = 0.95 Å for aryl and 0.98 A for methyl H atoms, *U*_iso_(H) = 1.2*U*_eq_ (C) for aryl and 1.5*U*_eq_ (C) for methyl H atoms.

### Crystal data

**Table:**

C_18_H_17_BrO_2_S	*Z* = 2
*M**_r_* = 377.29	*F*(000) = 384
Triclinic, *P*1	*D*_x_ = 1.566 Mg m^−^^3^
*a* = 7.4011 (2) Å	Mo *K*α radiation, λ = 0.71073 Å
*b* = 10.6609 (2) Å	Cell parameters from 7576 reflections
*c* = 11.1857 (2) Å	θ = 2.8–28.3°
α = 67.265 (1)°	µ = 2.70 mm^−^^1^
β = 86.593 (1)°	*T* = 173 K
γ = 79.384 (1)°	Block, colourless
*V* = 800.00 (3) Å^3^	0.60 × 0.54 × 0.48 mm

### Data collection

**Table:**

Bruker SMART APEXII CCD diffractometer	3982 independent reflections
Radiation source: rotating anode	3297 reflections with *I* > 2σ(*I*)
Detector resolution: 10.0 pixels mm^-1^	*R*_int_ = 0.046
φ and ω scans	θ_max_ = 28.4°, θ_min_ = 2.0°
Absorption correction: multi-scan (*SADABS*; Bruker, 2009)	*h* = −9→9
*T*_min_ = 0.269, *T*_max_ = 0.746	*k* = −14→14
14745 measured reflections	*l* = −14→14

### Refinement

**Table:**

Refinement on *F*^2^	Secondary atom site location: difference Fourier map
Least-squares matrix: full	Hydrogen site location: difference Fourier map
*R*[*F*^2^ > 2σ(*F*^2^)] = 0.035	H-atom parameters constrained
*wR*(*F*^2^) = 0.087	*w* = 1/[σ^2^(*F*_o_^2^) + (0.0371*P*)^2^ + 0.3816*P*] where *P* = (*F*_o_^2^ + 2*F*_c_^2^)/3
*S* = 1.05	(Δ/σ)_max_ = 0.001
3982 reflections	Δρ_max_ = 0.48 e Å^−^^3^
204 parameters	Δρ_min_ = −0.79 e Å^−^^3^
0 restraints	Extinction correction: *SHELXL2014* (Sheldrick 2014, Fc^*^=kFc[1+0.001xFc^2^λ^3^/sin(2θ)]^-1/4^
Primary atom site location: structure-invariant direct methods	Extinction coefficient: 0.040 (2)

### Special details

**Table:**

Experimental. ^1^H NMR (δ p.p.m., CDCl_3_, 400 Hz): 8.01 (d, J = 7.52 Hz, 1H), 7.35-7.44 (m, 2H), 7.15-7.23 (m, 2H), 2.64 (s, 3H), 2.53 (s, 3H), 2.50 (s, 3H), 2.15 (s, 3H).
Geometry. All esds (except the esd in the dihedral angle between two l.s. planes) are estimated using the full covariance matrix. The cell esds are taken into account individually in the estimation of esds in distances, angles and torsion angles; correlations between esds in cell parameters are only used when they are defined by crystal symmetry. An approximate (isotropic) treatment of cell esds is used for estimating esds involving l.s. planes.

### Fractional atomic coordinates and isotropic or equivalent isotropic displacement parameters (Å^2^)

**Table:**

	*x*	*y*	*z*	*U*_iso_*/*U*_eq_	
Br1	0.18479 (4)	0.88031 (3)	0.48841 (3)	0.04996 (12)	
S1	0.47494 (6)	0.56515 (5)	0.10695 (5)	0.02289 (12)	
O1	0.82901 (18)	0.52534 (15)	0.36828 (13)	0.0255 (3)	
O2	0.29324 (19)	0.52076 (15)	0.14556 (15)	0.0318 (3)	
C1	0.5830 (2)	0.5705 (2)	0.24007 (18)	0.0215 (4)	
C2	0.5307 (2)	0.63609 (19)	0.33233 (18)	0.0212 (4)	
C3	0.3713 (3)	0.7126 (2)	0.36069 (19)	0.0252 (4)	
C4	0.3916 (3)	0.7625 (2)	0.4560 (2)	0.0305 (5)	
C5	0.5531 (3)	0.7344 (2)	0.5293 (2)	0.0320 (5)	
C6	0.7045 (3)	0.6511 (2)	0.5047 (2)	0.0292 (4)	
H6	0.8151	0.6258	0.5538	0.035*	
C7	0.6896 (3)	0.6060 (2)	0.40654 (18)	0.0232 (4)	
C8	0.7605 (3)	0.5054 (2)	0.26755 (19)	0.0230 (4)	
C9	0.1912 (3)	0.7347 (2)	0.2948 (2)	0.0353 (5)	
H9A	0.0923	0.7254	0.3583	0.053*	
H9B	0.1951	0.6658	0.2564	0.053*	
H9C	0.1682	0.8275	0.2265	0.053*	
C10	0.5626 (4)	0.7902 (3)	0.6331 (2)	0.0456 (6)	
H10A	0.6826	0.7532	0.6771	0.068*	
H10B	0.4653	0.7624	0.6961	0.068*	
H10C	0.5460	0.8912	0.5936	0.068*	
C11	0.8919 (3)	0.4230 (2)	0.2093 (2)	0.0315 (5)	
H11A	0.8326	0.4174	0.1360	0.047*	
H11B	0.9303	0.3298	0.2745	0.047*	
H11C	0.9999	0.4674	0.1790	0.047*	
C12	0.4240 (3)	0.7459 (2)	0.00352 (18)	0.0237 (4)	
C13	0.2409 (3)	0.8030 (2)	−0.0253 (2)	0.0354 (5)	
H13	0.1482	0.7498	0.0148	0.042*	
C14	0.1930 (3)	0.9375 (3)	−0.1124 (3)	0.0458 (6)	
H14	0.0671	0.9775	−0.1315	0.055*	
C15	0.3276 (4)	1.0137 (3)	−0.1718 (2)	0.0425 (6)	
H15	0.2950	1.1065	−0.2312	0.051*	
C16	0.5101 (3)	0.9544 (2)	−0.1444 (2)	0.0356 (5)	
H16	0.6019	1.0078	−0.1858	0.043*	
C17	0.5636 (3)	0.8195 (2)	−0.05849 (19)	0.0266 (4)	
C18	0.7640 (3)	0.7562 (3)	−0.0369 (2)	0.0373 (5)	
H18A	0.8362	0.8142	−0.1052	0.056*	
H18B	0.7826	0.6637	−0.0391	0.056*	
H18C	0.8037	0.7494	0.0478	0.056*	

### Atomic displacement parameters (Å^2^)

**Table:**

	*U*^11^	*U*^22^	*U*^33^	*U*^12^	*U*^13^	*U*^23^
Br1	0.05418 (19)	0.03685 (17)	0.05438 (19)	0.00746 (11)	0.01541 (12)	−0.02160 (13)
S1	0.0205 (2)	0.0245 (3)	0.0253 (2)	−0.00667 (18)	−0.00178 (17)	−0.0098 (2)
O1	0.0211 (7)	0.0289 (8)	0.0257 (7)	−0.0014 (5)	−0.0027 (5)	−0.0104 (6)
O2	0.0237 (7)	0.0313 (8)	0.0385 (8)	−0.0127 (6)	−0.0026 (6)	−0.0074 (7)
C1	0.0181 (8)	0.0234 (10)	0.0229 (9)	−0.0051 (7)	0.0006 (7)	−0.0081 (8)
C2	0.0211 (9)	0.0195 (9)	0.0206 (9)	−0.0045 (7)	0.0020 (7)	−0.0047 (7)
C3	0.0254 (9)	0.0205 (10)	0.0240 (10)	−0.0038 (7)	0.0041 (7)	−0.0028 (8)
C4	0.0375 (11)	0.0194 (10)	0.0291 (11)	−0.0006 (8)	0.0082 (8)	−0.0062 (8)
C5	0.0500 (13)	0.0205 (10)	0.0221 (10)	−0.0050 (9)	0.0016 (9)	−0.0051 (8)
C6	0.0370 (11)	0.0247 (11)	0.0234 (10)	−0.0047 (8)	−0.0046 (8)	−0.0059 (8)
C7	0.0245 (9)	0.0200 (10)	0.0220 (9)	−0.0033 (7)	0.0003 (7)	−0.0047 (8)
C8	0.0208 (9)	0.0244 (10)	0.0241 (10)	−0.0058 (7)	0.0003 (7)	−0.0089 (8)
C9	0.0229 (10)	0.0347 (12)	0.0435 (13)	0.0015 (8)	0.0022 (9)	−0.0128 (10)
C10	0.0743 (18)	0.0328 (13)	0.0322 (13)	−0.0039 (12)	−0.0018 (12)	−0.0173 (11)
C11	0.0218 (9)	0.0345 (12)	0.0386 (12)	0.0011 (8)	0.0011 (8)	−0.0172 (10)
C12	0.0271 (9)	0.0242 (10)	0.0205 (9)	−0.0055 (8)	−0.0020 (7)	−0.0086 (8)
C13	0.0252 (10)	0.0353 (12)	0.0372 (12)	−0.0051 (9)	−0.0058 (9)	−0.0040 (10)
C14	0.0367 (13)	0.0393 (14)	0.0466 (15)	0.0009 (10)	−0.0116 (11)	−0.0022 (11)
C15	0.0552 (15)	0.0263 (12)	0.0369 (13)	−0.0047 (11)	−0.0060 (11)	−0.0022 (10)
C16	0.0467 (13)	0.0309 (12)	0.0300 (11)	−0.0169 (10)	0.0021 (9)	−0.0082 (9)
C17	0.0299 (10)	0.0317 (11)	0.0232 (10)	−0.0108 (8)	0.0013 (8)	−0.0136 (9)
C18	0.0287 (11)	0.0438 (14)	0.0376 (12)	−0.0146 (10)	0.0073 (9)	−0.0110 (10)

### Geometric parameters (Å, º)

**Table:**

Br1—C4	1.905 (2)	C9—H9C	0.9800
Br1—Br1^i^	3.4521 (5)	C10—H10A	0.9800
S1—O2	1.4921 (14)	C10—H10B	0.9800
S1—C1	1.7561 (19)	C10—H10C	0.9800
S1—C12	1.805 (2)	C11—H11A	0.9800
O1—C8	1.366 (2)	C11—H11B	0.9800
O1—C7	1.376 (2)	C11—H11C	0.9800
C1—C8	1.360 (3)	C12—C13	1.381 (3)
C1—C2	1.454 (3)	C12—C17	1.399 (3)
C2—C7	1.395 (3)	C13—C14	1.381 (3)
C2—C3	1.399 (3)	C13—H13	0.9500
C3—C4	1.389 (3)	C14—C15	1.377 (4)
C3—C9	1.498 (3)	C14—H14	0.9500
C4—C5	1.409 (3)	C15—C16	1.379 (4)
C5—C6	1.381 (3)	C15—H15	0.9500
C5—C10	1.505 (3)	C16—C17	1.384 (3)
C6—C7	1.375 (3)	C16—H16	0.9500
C6—H6	0.9500	C17—C18	1.504 (3)
C8—C11	1.481 (3)	C18—H18A	0.9800
C9—H9A	0.9800	C18—H18B	0.9800
C9—H9B	0.9800	C18—H18C	0.9800
			
C4—Br1—Br1^i^	173.50 (6)	C5—C10—H10B	109.5
O2—S1—C1	110.19 (9)	H10A—C10—H10B	109.5
O2—S1—C12	105.80 (9)	C5—C10—H10C	109.5
C1—S1—C12	101.64 (9)	H10A—C10—H10C	109.5
C8—O1—C7	106.53 (14)	H10B—C10—H10C	109.5
C8—C1—C2	106.98 (16)	C8—C11—H11A	109.5
C8—C1—S1	117.93 (15)	C8—C11—H11B	109.5
C2—C1—S1	135.02 (15)	H11A—C11—H11B	109.5
C7—C2—C3	119.21 (18)	C8—C11—H11C	109.5
C7—C2—C1	104.32 (16)	H11A—C11—H11C	109.5
C3—C2—C1	136.46 (18)	H11B—C11—H11C	109.5
C4—C3—C2	115.32 (18)	C13—C12—C17	121.35 (19)
C4—C3—C9	122.90 (19)	C13—C12—S1	116.73 (15)
C2—C3—C9	121.76 (19)	C17—C12—S1	121.36 (15)
C3—C4—C5	125.3 (2)	C14—C13—C12	119.8 (2)
C3—C4—Br1	117.17 (16)	C14—C13—H13	120.1
C5—C4—Br1	117.52 (16)	C12—C13—H13	120.1
C6—C5—C4	117.8 (2)	C15—C14—C13	120.0 (2)
C6—C5—C10	120.1 (2)	C15—C14—H14	120.0
C4—C5—C10	122.0 (2)	C13—C14—H14	120.0
C7—C6—C5	117.64 (19)	C14—C15—C16	119.6 (2)
C7—C6—H6	121.2	C14—C15—H15	120.2
C5—C6—H6	121.2	C16—C15—H15	120.2
C6—C7—O1	124.63 (17)	C15—C16—C17	122.1 (2)
C6—C7—C2	124.46 (19)	C15—C16—H16	119.0
O1—C7—C2	110.91 (16)	C17—C16—H16	119.0
C1—C8—O1	111.23 (17)	C16—C17—C12	117.10 (19)
C1—C8—C11	133.54 (19)	C16—C17—C18	120.40 (19)
O1—C8—C11	115.20 (16)	C12—C17—C18	122.48 (19)
C3—C9—H9A	109.5	C17—C18—H18A	109.5
C3—C9—H9B	109.5	C17—C18—H18B	109.5
H9A—C9—H9B	109.5	H18A—C18—H18B	109.5
C3—C9—H9C	109.5	C17—C18—H18C	109.5
H9A—C9—H9C	109.5	H18A—C18—H18C	109.5
H9B—C9—H9C	109.5	H18B—C18—H18C	109.5
C5—C10—H10A	109.5		
			
O2—S1—C1—C8	130.07 (16)	C3—C2—C7—C6	−2.7 (3)
C12—S1—C1—C8	−118.10 (16)	C1—C2—C7—C6	178.32 (19)
O2—S1—C1—C2	−53.3 (2)	C3—C2—C7—O1	177.36 (16)
C12—S1—C1—C2	58.6 (2)	C1—C2—C7—O1	−1.6 (2)
C8—C1—C2—C7	1.7 (2)	C2—C1—C8—O1	−1.1 (2)
S1—C1—C2—C7	−175.25 (16)	S1—C1—C8—O1	176.39 (13)
C8—C1—C2—C3	−177.1 (2)	C2—C1—C8—C11	−179.1 (2)
S1—C1—C2—C3	6.0 (4)	S1—C1—C8—C11	−1.6 (3)
C7—C2—C3—C4	5.3 (3)	C7—O1—C8—C1	0.1 (2)
C1—C2—C3—C4	−176.1 (2)	C7—O1—C8—C11	178.51 (17)
C7—C2—C3—C9	−173.01 (18)	O2—S1—C12—C13	−3.79 (19)
C1—C2—C3—C9	5.6 (3)	C1—S1—C12—C13	−118.91 (17)
C2—C3—C4—C5	−4.3 (3)	O2—S1—C12—C17	−175.31 (16)
C9—C3—C4—C5	174.0 (2)	C1—S1—C12—C17	69.58 (17)
C2—C3—C4—Br1	174.84 (13)	C17—C12—C13—C14	−3.1 (3)
C9—C3—C4—Br1	−6.9 (3)	S1—C12—C13—C14	−174.6 (2)
C3—C4—C5—C6	0.3 (3)	C12—C13—C14—C15	1.0 (4)
Br1—C4—C5—C6	−178.87 (15)	C13—C14—C15—C16	0.5 (4)
C3—C4—C5—C10	−178.6 (2)	C14—C15—C16—C17	0.0 (4)
Br1—C4—C5—C10	2.2 (3)	C15—C16—C17—C12	−2.0 (3)
C4—C5—C6—C7	2.7 (3)	C15—C16—C17—C18	176.6 (2)
C10—C5—C6—C7	−178.4 (2)	C13—C12—C17—C16	3.5 (3)
C5—C6—C7—O1	178.41 (18)	S1—C12—C17—C16	174.66 (16)
C5—C6—C7—C2	−1.5 (3)	C13—C12—C17—C18	−175.1 (2)
C8—O1—C7—C6	−178.95 (19)	S1—C12—C17—C18	−3.9 (3)
C8—O1—C7—C2	1.0 (2)		

Symmetry code: (i) −*x*, −*y*+2, −*z*+1.

### Hydrogen-bond geometry (Å, º)

Cg1 is the centroid of the C2–C7 benzene ring.

**Table:**

*D*—H···*A*	*D*—H	H···*A*	*D*···*A*	*D*—H···*A*
C9—H9*B*···O2	0.98	2.33	3.274 (3)	162
C11—H11*C*···O2^ii^	0.98	2.32	3.276 (2)	166
C15—H15···*Cg*1^iii^	0.95	2.88	3.659 (3)	140

Symmetry codes: (ii) *x*+1, *y*, *z*; (iii) −*x*+1, −*y*+1, −*z*+1.
