# Supplementary figures and images for: Crystal structure of 5-bromo-2,4,6-trimethyl-3-[(2-methyl­phen­yl)sulfin­yl]-1-benzo­furan
Source: Acta Crystallogr E Crystallogr Commun. 2015 Jul 25;71(Pt 8):o602–3. doi: 10.1107/S2056989015013687 (PMC4571419; doi:10.1107/S2056989015013687)

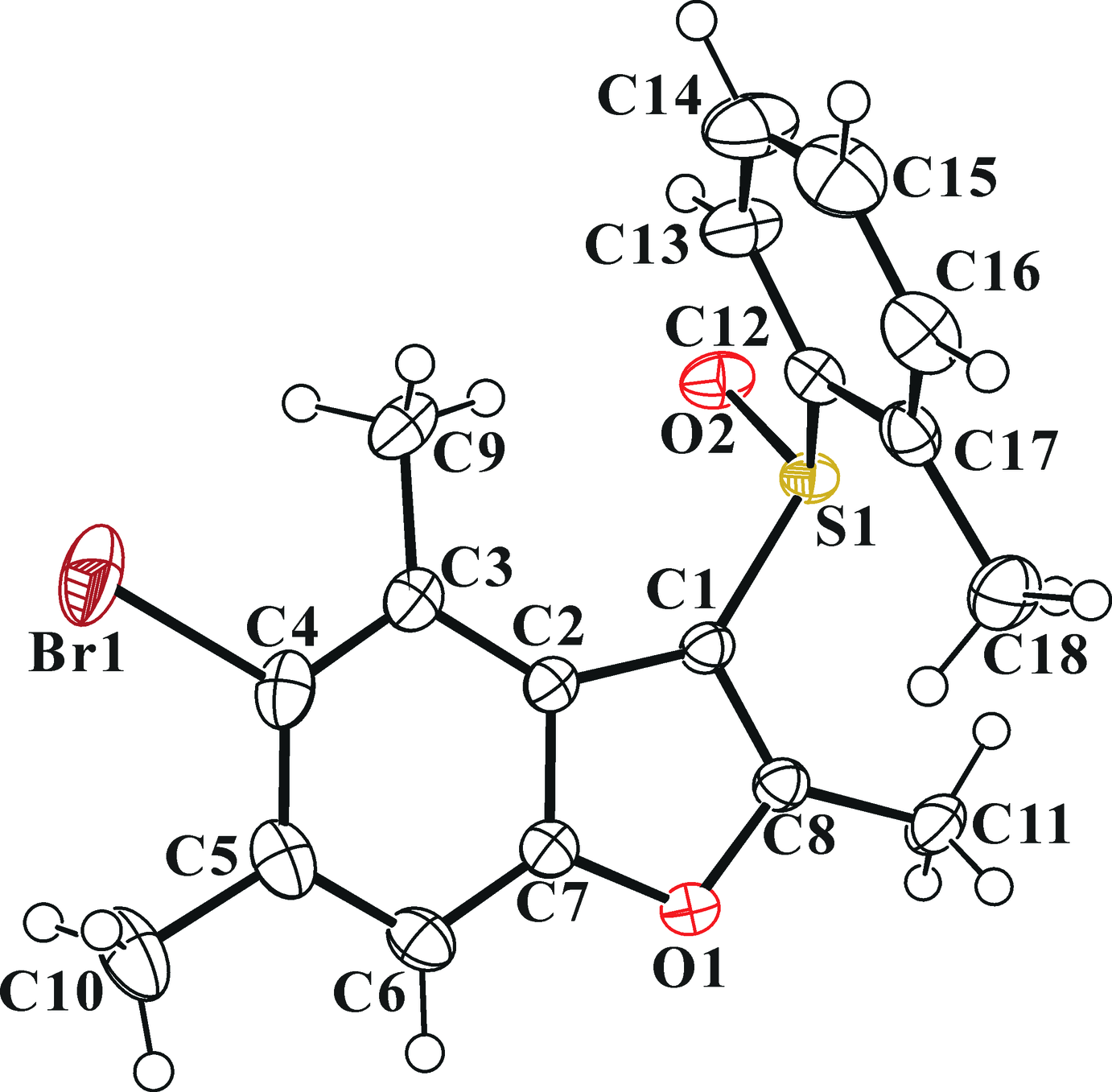

Supplement: Supplementary file 4 [file e-71-0o602-fig1.tif]

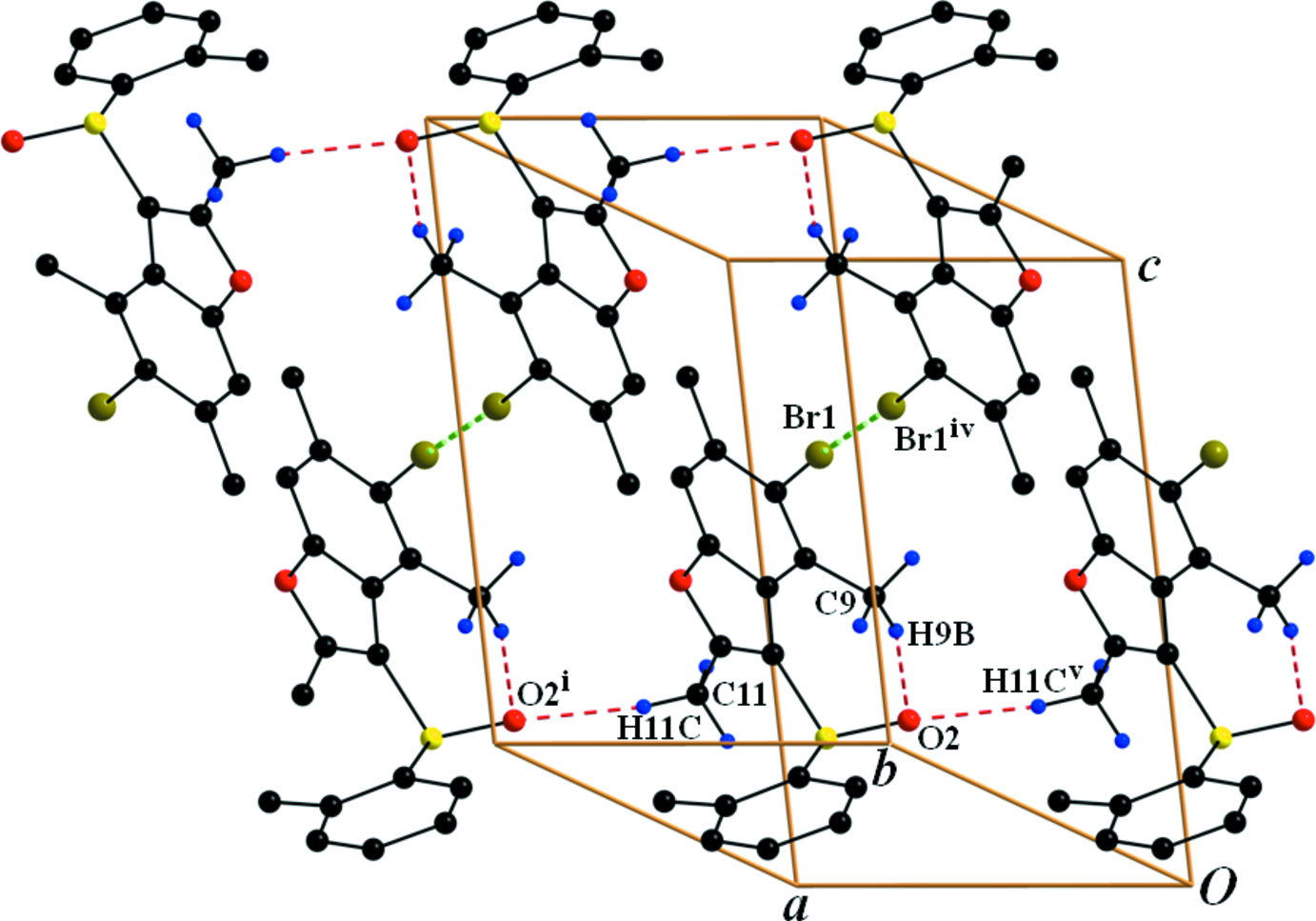

Supplement: Supplementary file 5 [file e-71-0o602-fig2.tif]

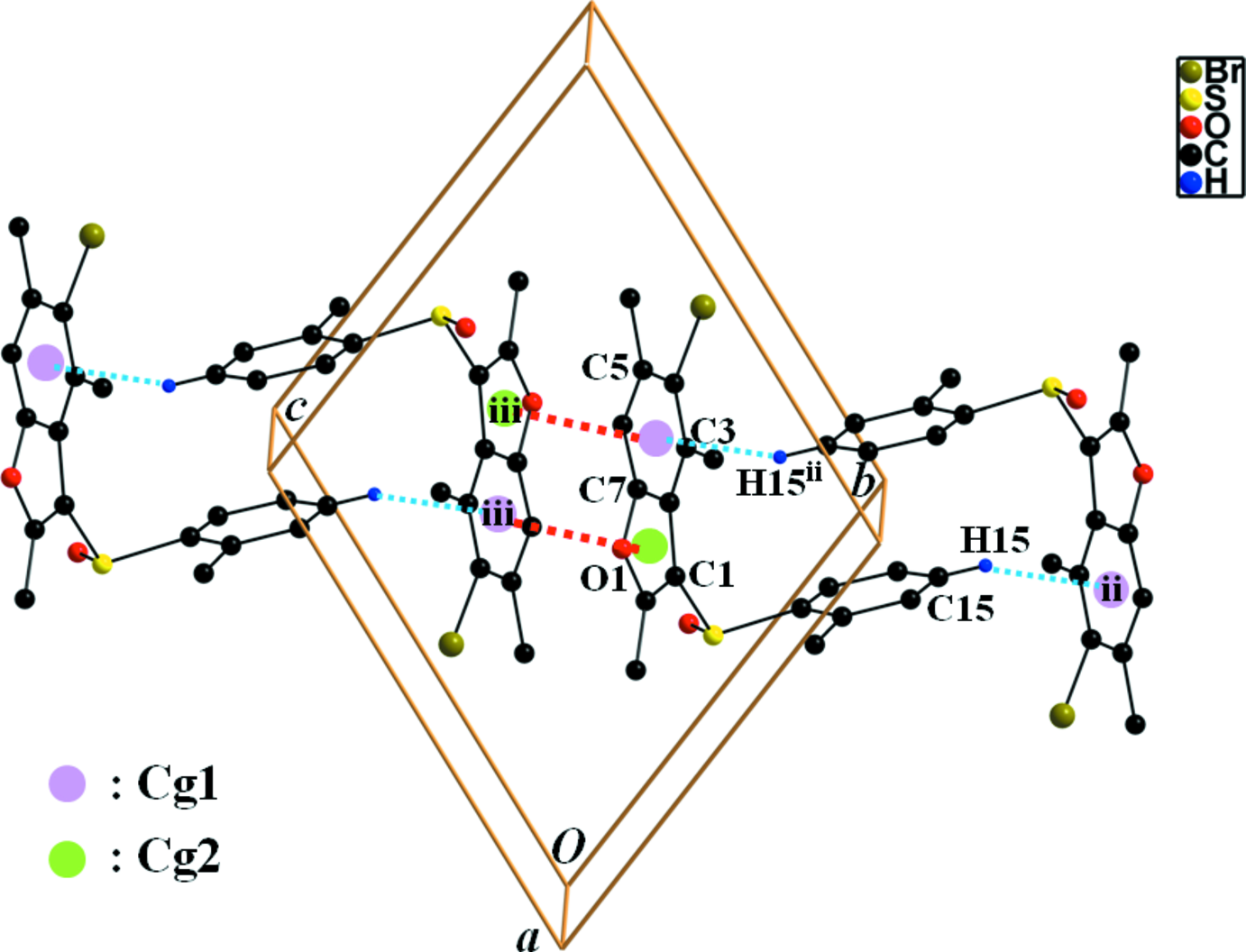

Supplement: Supplementary file 6 [file e-71-0o602-fig3.tif]
